# Therapeutic effects of lentivirus-mediated shRNA targeting of cyclin D1 in human gastric cancer

**DOI:** 10.1186/1471-2407-14-175

**Published:** 2014-03-11

**Authors:** Jin-Hee Seo, Eui-Suk Jeong, Yang-Kyu Choi

**Affiliations:** 1 Department of Laboratory Animal Medicine, College of Veterinary Medicine, Konkuk University, Neungdong-ro, Gwangjin-gu, Seoul 143-701, Republic of Korea; 2 Veterinary Science Research Institute, College of Veterinary Medicine, Konkuk University, Neungdong-ro, Gwangjin-gu, Seoul 143-701, Republic of Korea; 3 Laboratory Animal Facility, Korea Institute of Radiological and Medical Sciences, Seoul 139-706, Republic of Korea

**Keywords:** Gastric cancer, Cyclin D1, Lentivirus, shRNA

## Abstract

**Background:**

Gastric cancer is the second most common cause of cancer-related death in males and the fourth in females. Traditional treatment has poor prognosis because of recurrence and systemic side effects. Therefore, the development of new therapeutic strategies is an important issue. Lentivirus-mediated shRNA stably inhibits target genes and can efficiently transduce most cells. Since overexpressed cyclin D1 is closely related to human gastric cancer progression, inhibition of cyclin D1 using specific targeting could be an effective treatment method of human gastric cancer.

**Methods:**

The therapeutic effect of lentivirus-mediated shRNA targeting of cyclin D1 (ShCCND1) was analyzed both *in vitro* and *in vivo* experiments.

**Results:**

*In vitro*, NCI-N87 cells with downregulation of cyclin D1 by ShCCND1 showed significant inhibition of cell proliferation, cell motility, and clonogenicity. Downregulation of cyclin D1 in NCI-N87 cells also resulted in significantly increased G1 arrest and apoptosis. *In vivo*, stable NCI-N87 cells expressing ShCCND1 were engrafted into nude mice. Then, the cancer-growth inhibition effect of lentivirus was confirmed. To assess lentivirus including ShCCND1 as a therapeutic agent, intratumoral injection was conducted. Tumor growth of the lentivirus-treated group was significantly inhibited compared to growth of the control group. These results are in accordance with the *in vitro* data and lend support to the mitotic figure count and apoptosis analysis of the tumor mass.

**Conclusion:**

The lentivirus-mediated ShCCND1 was constructed, which effectively inhibited growth of NCI-N87-derived cancer both *in vitro* and *in vivo*. The efficiency of shRNA knockdown and variation in the degree of inhibition is mediated by different shRNA sequences and cancer cell lines. These experimental results suggest the possibility of developing new gastric cancer therapies using lentivirus-mediated shRNA.

## Background

Gastric cancer is the one of the most common causes of cancer-related death all over the world. The survival of gastric cancer patients has improved owing to improvement in diagnostic and therapeutic tools [1]. However, overall patient prognosis and long-term survival has not significantly improved due to diagnosis of advanced stages and the high rate of recurrence [2]. In addition, the biologic heterogeneity of gastric cancer is one of the main causes of cancer therapy failure [3]. Therefore, it is necessary to find a novel strategy to overcome gastric cancer.

Cyclin D1, a cell cycle regulator, is closely related to several human cancers. Dysregulation of cyclin D1 immediately contributes to tumor progression in some animal models, further demonstrating a connection between cyclin D1 and carcinogenesis. Accordingly, inhibition of cyclin D1 may be an innovative strategy for cancer treatment [4,5].

Targeted therapy is becoming an important cancer therapy. Although gene targeting has emerged as an important treatment strategy of various malignant tumors in the past decade, fewer targeted therapies for gastric cancer have been studied compared to other cancers [6]. RNAi is one of the most powerful targeted therapies to inhibit specific gene expression at the post-transcriptional level. There are two types of artificial RNAi induction: siRNA and shRNA. Previous reports have demonstrated the following limitations of siRNA: insufficient cellular uptake, instability, rapid clearance *in vitro*, and inefficient delivery *in vivo* and in clinical settings [7-10]. However, viral vector-mediated shRNA permit long-term stable and consistent gene silencing [11]. Lentiviral vectors of shRNA have been derived from HIV; the vectors can express shRNA in mammalian cells [12,13]. These vectors have been validated in clinical databases in animal models of relevant cancers. Therefore, the lentivirus-mediated shRNA strategy is an appealing candidate therapy for cancer treatment [14].

In this study, we hypothesized that inhibition of cyclin D1 in human gastric cancer could suppress cancer progression *in vitro* and *in vivo*. To test this hypothesis, a third-generation lentiviral vector was used to integrate the shRNA sequence targeting cyclin D1 (ShCCND1) in the human gastric cancer cell line NCI-N87. ShRNA was expressed stably and consistently inhibited cyclin D1 expression. *In vitro,* the effect of silencing cyclin D1 on gastric cancer cell function, including cell proliferation, cell cycle, apoptosis, and cell motility was examined. *In vivo*, stably ShCCND1-expressing NCI-N87 xenografts were used to compare the growth rate with ShSramble (shRNA-targeting scramble) cell xenografts. Next, the effect of intratumoral injection of these recombinant lentiviruses targeting cyclin D1 on attenuation of the growth of pre-existing cancer was assessed.

## Methods

### Cell lines and cell culture

NCI-N87 gastric carcinoma cells were purchased from the Korean Cell Line Bank (KCLB, Seoul, Korea). Cells were maintained in RPMI 1640 with 10% FBS and 1% penicillin/streptomycin at 37°C, 5% CO_2_ within a humidified chamber. The 293TN Human Kidney producer cell line was purchased from System Biosciences (SBI, Mountain View, CA, USA). This cell line was maintained in DMEM high-glucose medium supplemented with 10% FBS, Glutamax, and 1% penicillin/streptomycin at 37°C, 5% CO_2_ within a humidified chamber.

### ShRNA-expressing plasmid DNA

Three different targeted sequences designed to be homologous to cyclin D1 were designed using the lentiviral expression vector (pGreenPuro™ Vector, SBI) [15]. Target sites in human genes encoding cyclin D1 were as follows: ShCCND1_1 sense strand, 5′-GCCCTCGGTGTCCTACTTCAAAT-3′, ShCCND1_1 antisense strand, 5′-ATTTGAAGTAGGACACCGAGGGC-3′; ShCCND1_2 sense strand, 5′-GCACGATT TCATTGAACACTTCC-3′, ShCCND1_2 antisense strand, 5′- GGAAGTGT TCAATGAAATCGTGC-3′; ShCCND1_3 sense strand, 5′-GAAGTTCATTTCCAATCCGCCCT-3, ShCCND1_3 antisense strand, 5′-AGGGCGGATTGGAAATGAACTTC-3′. These plasmid DNAs transcribed shRNA with loop sequences of 5′-CTTCCTGTCAGA-3′. A negative control sequence was also designed by the same process, which had no homology with human proteins (ShScramble sense strand, 5′-GACTTCATAAGGC GCATGC-3′, ShScramble antisense strand, 5′-GCATGCGCCT TATGAAGTC-3′) [16]. Each DNA was used to transform the *E. coli* strain DH5α and purified with a plasmid purification kit (Qiagen, Valencia, CA, USA). The ligation product was confirmed by PCR and sequencing.

### Lentivirus generation and establishment of the NCI-N87 cell line stably expressing shRNA

293TN cells were seeded at a density of 3 × 10^6^ cells on 10-cm culture plates. After 24 h, successful co-transfection of plasmid including the ShCCND1 or ShScramble with lentiviral vector expression construct using lipofectamine reagent was demonstrated. After 48 h, supernatant was collected and added to PEG-*it*™ virus precipitation solution. The supernatant/PEG-*IT* mixture was centrifuged 1,500 × *g* for 30 min at 4°C and then resuspended in RPMI medium at 1/100 of the original volume. One day prior to transduction, NCI-N87 cells were plated in 24-well plates at 5 × 10^4^ cells. After 24 h, NCI-N87 cells were infected with lentiviral particles containing ShScramble or ShCCND1. The next day, NCI-N87 cells were cultured in RPMI 1640 medium including puromycin (1 μg/ml). The expanded cells were then used for further experiments. Transduction efficiency of NCI-N87 cells with GFP signals were determined by flow cytometry.

### Western blotting

Stable cancer cells (NCI-N87, ShScramble, and ShCCND1) were lysed in RIPA buffer including protease inhibitor (Sigma-Aldrich, St. Louis, MO, USA). The protein concentration of cell lysate was determined using the BCA™ protein assay kit (Thermo Scientific, Rockford, IL, USA). Equal amounts of total protein were electrophoresed by 10% SDS-PAGE, transferred onto nitrocellulose membranes, and incubated with antibodies against cyclin D1 (1:500, Santa-Cruz Biotechnology, Santa Cruz, CA, USA), pRB (1:200, Cell Signaling Technology, Beverly, MA, USA), and β-actin (1:500, Santa-Cruz Biotechnology) at 4°C. Membranes were then incubated with HRP-conjugated anti-rabbit or anti-mouse secondary antibodies (1:1000, Santa-Cruz Biotechnology). Signals were detected using an ECL Test Kit (KPL, Gaithersburg, MD, USA). β-actin served as the internal standard. Densitometric analysis was performed using ImageJ software.

### Cell proliferation assay and colony formation assay

The *in vitro* cell viability was analyzed using the CCK-8 assay (Dojindo Laboratories, Kumamoto, Japan). NCI-N87, ShScramble, and ShCCND1 were seeded at a density of 10^4^ cells/well in 96-well plates. After 24 h, 10 μl of CCK-8 was added to each well and incubated for 2 h. The absorbance value was observed at 1, 2, 3, and 4 days using an enzyme-linked immunosorbent assay (Tecan Sunrise, Sunnyville, CA, USA).

To assess colony formation ability, NCI-N87 cells containing shRNA were seeded at a density of 500 cells/well in 6-well plates. After 3 weeks, cells were stained with 1% crystal violet. The number of colonies (≥25 cells) was counted under a microscope. The relative number of colonies in ShCCND1 was adjusted to the number in ShScramble. Image analysis was conducted using Metamorph version 7.5.6.0 software (Molecular Devices, Sunnyvale, CA, USA).

### Scratch-wound healing assay

The scratch-wound healing assay was performed to determine the role of cyclin D1 in cell migration [17]. NCI-N87 cells were plate on 60-mm plates at 5 × 10^4^ cells. A scratch was made with a pipette tip when the plate was almost filled with cells. After 48 h, the image of cells that had migrated into the wounded area was obtained by a Zeiss Axiovert 200 inverted microscope (Carl Zeiss MicroImaging, Thornwood, NY, USA) with a 10× objective lens. The wound area was analyzed by Metamorph version 7.5.6.0 software.

### Cell cycle and apoptosis analysis

To determine the cell cycle distribution, 10^6^ NCI-N87 cells were washed twice with ice-cold PBS and resuspended in PBS containing RNase A (Sigma-Aldrich) and PI (propidium iodide, Sigma-Aldrich). Cells were incubated at 37°C in the dark for 1 h. The percentage of the cell population in each phase of the cell cycle was measured using FACSCalibur (Becton-Dickinson, Rutherford, NJ, USA), and results were analyzed with the CELLQUEST software (Becton-Dickinson).

To assess the apoptosis rate, NCI-N87 cells were starved in FBS-free culture medium for 48 h. Then, aliquots (100 μl) of 10^6^ cells/ml were incubated with Annexin-V-APC (allophycocyanin, BD Biosciencls Pharmingen, San Diego, CA, USA) and PI (BD Biosciences Pharmingen) for 15 min in the dark. After incubation, 400 μl of the binding buffer was added to the sample and then, samples were analyzed by flow cytometry. The flow cytometric data were analyzed with the CELLQUEST software.

### Tumorigenecity in nude mouse

Male athymic BALB/c nude mice at 5 weeks of age were purchased from the Nara Bio animal center (NARA Biotech, Seoul, Korea). The mice were bred and maintained at the laboratory facility in Konkuk University (Seoul, Korea). Tumorigenicity in nude mice was determined. The NCI-N87 cell line including ShScramble or ShCCND1 was cultured with RPMI 1640 medium. Cells (8 × 10^6^) in 100 μl saline were subcutaneously injected into the posterior right thigh. Tumor growth was measured by the tumor diameter with a digital vernier caliper three times a week from the 9th day until the 35th day after tumor injection. Tumor volume (mm^3^) was calculated according to the following equation: tumor volume = d^2^ × D/2, where d and D represent the shortest and the longest diameter, respectively. At the end of the experiment (the 35th day), mice were sacrificed. Tumorigenecity study was approved by the IACUC (Institutional Animal Care and Use Committee) of Konkuk University (KU12036).

### Titration and intratumoral injection of lentivirus-mediated ShCCND1 into established tumors

NCI-N87 cells were seeded at a density of 4 × 10^5^ cells per well in 6-well plates. After 24 h, cell culture medium was replaced by RPMI 1640 medium including hexadimethrine bromide. Then, lentiviral stock in serial dilution was added to pre-plated NCI-N87 cells. After 48 h incubation, analysis was performed by flow cytometer. GFP-positive NCI-N87 cells were detected by flow cytometry. Then, the virus titer was calculated by the following equation: lentivirus titer in IU (infectious unit)/ml = {(cell number at starting time) × (dilution factor) × (percent infection)}/(added virus volume expressed in ml).

To assess the inhibition effects of virus particle including ShCCND1 on established tumors, male athymic BALB/c nude mice (NARA Biotech, N = 20) at 5 weeks of age were injected subcutaneously into the posterior right thigh with 100 μl saline containing 4 × 10^6^ NCI-N87 cells. The mice were divided randomly into three groups. Once tumors reached a volume of 80–100 mm^3^ (usually 6–8 days after injection of the cells), mice received an intratumoral injection of 50 μl (4 × 10^7^ IU/ml) of saline (Saline, N = 6), virus containing ShScramble (Virus_Scramble, N = 7), or virus containing ShCCND1 (Virus_CCND1, N = 7). After 1 week, Saline, Virus_Scramble, or Virus_CCND1 was reinjected into the tumor mass. *In vivo* therapeutic study was approved by the IACUC of Konkuk University (KU13049).

### H&E and TUNEL staining

At day 13 after the first intratumoral injection, the mice were sacrificed and subcutaneous tumors were fixed in 10% neutral-buffered formalin. Fixed tumors were routinely processed for paraffin sectioning and H&E staining for histopathological examination. For quantification of the mitotic count, mitotic figures were counted in five random high-fields (400×) per slide and expressed as the average number of mitotic figures per field. The detection of apoptosis was performed according to the instructions of the manufacturer (ApopTag, Chemicon International Inc., Temecula, CA, USA). Briefly, the sections were deparaffinized and hydrated, and then treated with proteinase K (20 μg/ml) in PBS (pH 7.5) at 37°C for 20 min followed by treatment with 3% hydroperoxidase in PBS to block endogenous peroxidases. After washing with PBS, the slides were incubated with terminal deoxynucleotidyl transferase and nucleotide mixture at 37°C for 1 h, and then anti-digoxigenin conjugate was added for 30 min. The slides were treated with DAB before counterstaining with hematoxylin. To measure the number of apoptotic cells, five fields at 400× magnification per slide were quantitatively analyzed with the image analyzer (Metamorph version 7.5.6.0 software). The data were expressed as the average number of cells per field.

### Statistical analysis

For statistical analysis, all data obtained were analyzed using Prism 5 for Windows software (GraphPad Software, San Diego, USA). Statistically significant differences between studied groups were evaluated using the unpaired Student’s *t*-test and Fisher’s exact test. The results were determined to be significantly significant when P < 0.05 was obtained.

## Results

### ShCCND1 significantly inhibited cyclin D1 expression and cell proliferation in NCI-N87 cells

**Figure 1:**
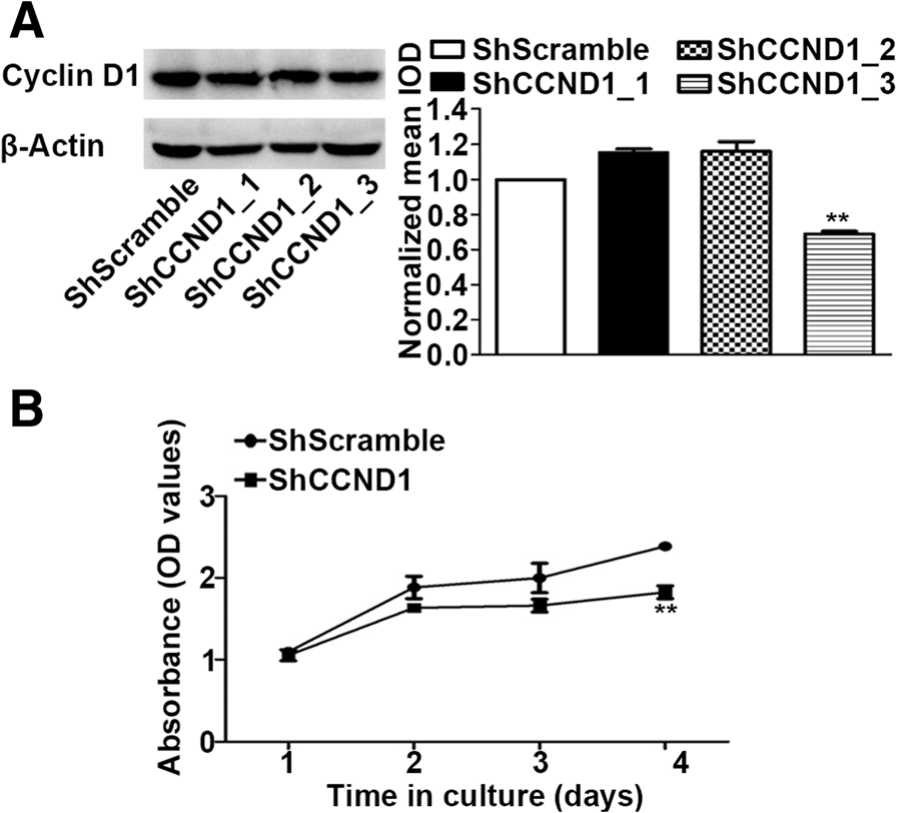
**Effect of ShCCND1 on cyclin D1 expression and cell proliferation in NCI-N87 cells.** Expression level of cyclin D1 protein in ShCCND1_3 was significantly inhibited in AGS cells by western blotting **(A)**. The viability of ShCCND1 demonstrated significant inhibition of proliferation in NCI-N87 cells compared to inhibition by ShScramble using the CCK-8 assay **(B)**. The results presented are the mean ± SEM. **P < 0.01 versus the ShScramble-transduced cells.

NCI-N87 cells infected with lentivirus were successfully selected in medium containing puromycin (1 μg/ml). The transduction efficiency of each cell line was 97.41% (ShScramble) and 95.8% (ShCCND1) by flow cytometry (data not shown). Western blotting was performed to assess the role of ShCCND1 in cyclin D1 expression levels. There was no difference in cyclin D1 expression levels between NCI-N87 cells and ShScramble cells (data not shown). In comparison with control, ShCCND1_1 and ShCCND1_2 did not decrease protein levels of cyclin D1 (Figure 1A). In contrast, ShCCND1_3 resulted in decreased protein levels compared to NCI-N87 (31%). Densitometric analysis of western blots identified a significant (P < 0.01) decrease in ShCCND1_3 in NCI-N87 cells. The ShCCND1_3 cells were then used for subsequent studies and referred to as ShCCND1. The expression level of pRB, a downstream molecule of cyclin D1, showed significant inhibition in ShCCND1 (data not shown). These data suggest that lentivirus-mediated ShCCND1 could effectively inhibit endogenous cyclin D1 expression in NCI-N87 cells. To assess the inhibitory effect of silencing cyclin D1 on cell proliferation, cell viability was analyzed using the CCK-8 assay. ShCCND1 exhibited dramatic inhibition of proliferation in NCI-N87 cells compared to ShScramble cells (Figure 1B). The suppression of proliferation by ShCCND1 was more significant at day 4 (P < 0.01). The viability of ShCCND1 was lower than ShScramble during the experiment. These results confirmed that ShCCND1 inhibited proliferation of the NCI-N87 human gastric cancer cells.

### ShCCND1 inhibited colony formation and cell motility in NCI-N87 cells

**Figure 2:**
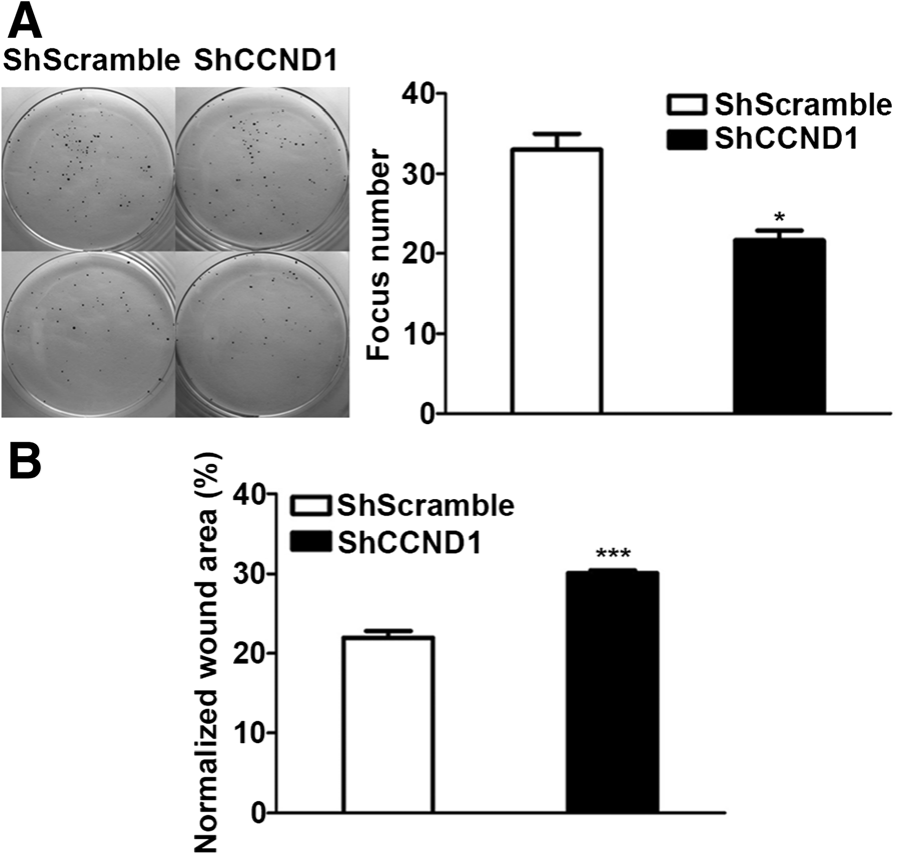
**Colony formation and scratch-wound healing assay.** Colony formation assays of ShCCND1 revealed a decrease in colony number compared to that of ShScramble **(A)**. The wound area of ShCCND1 was significantly increased compared to the wound area of ShScramble measured by the scratch-wound healing assay **(B)**. The data presented are the mean ± SEM. ***P < 0.001 versus the ShScramble-transduced cells.

The colony formation assay was performed to test whether ShCCND1 affected clonogenic potential, which is an important characteristic of tumor growth *in vivo*[18]. As shown in Figure 2A, ShCCND1 (N = 21.7) resulted in smaller colony numbers compared to ShScramble (N = 33.0, P < 0.05). These results indicate that silencing cyclin D1 may prevent cell proliferation and indicate that inhibition of cyclin D1 significantly decreased the colony formation potential of cells, which correlates with the formation of cancer in nude mouse [19]. The motility of NCI-N87 cells was analyzed by the scratch-wound healing assay because decreased clonogenic potential is usually associated with invasion ability in cancer cells [18]. In this assay, ShCCND1 showed a wider wound area compared to ShScramble (Figure 2B). The wound area was 30.1% and 21.9% in ShCCND1 and ShScramble, respectively. The wound area of ShCCND1 was significantly decreased compared to ShScramble (P < 0.001). This result indicates that ShCCND1 cells have impaired migration capacity.

### ShCCND1 affected cell cycle distribution and the apoptosis rate

**Figure 3:**
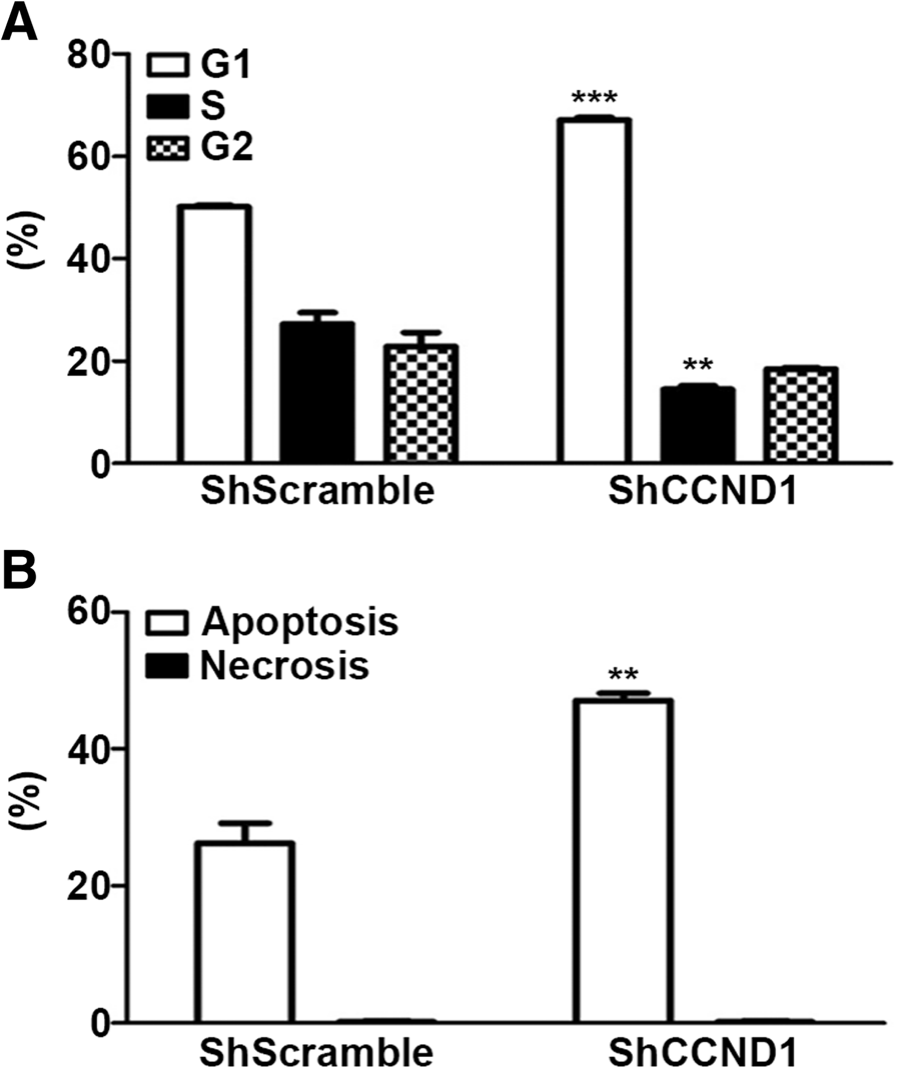
**Effect of ShCCND1 on NCI-N87 cell cycle distribution and apoptosis determined by flow cytometry.** ShCCND1 significantly increased the G1 distribution of the cell cycle compared to that of ShScramble **(A)**. Apoptosis was significantly increased in ShCCND1 compared to that of ShScramble **(B)**. The data presented are the mean ± SEM. **P < 0.01 and ***P < 0.001 versus the ShScramble-transduced cells.

To identify the mechanism for the anti-proliferation effect, cell cycle distribution of ShCCND1 was analyzed. As shown in Figure 3A, ShCCND1 induced a significant G1 arrest in comparison with ShScramble (P < 0.001) due to a reduction in the number of S phase cells (P = 0.058), while there was little difference in the number of cells in G2 phase. The G1 phase distribution of ShCCND1 was 67.2% in comparison with 50.17% in ShScramble. These results suggest that ShCCND1 inhibits cell proliferation by inducing Gl phase arrest. In addition to cell cycle arrest, the rate of cellular apoptosis was examined by flow cytometry. As shown in Figure 3B, ShCCND1 cells resulted in a significant enhancement in apoptosis compared to NCI-N87 cells. The cellular apoptosis rate was 29.19% in ShScramble and 48.14% in ShCCND1. Therefore, knockdown of cyclin D1 significantly reduced apoptotic cell death in NCI-N87 cells (P < 0.01).

### ShCCND1 suppressed xenograft tumor growth *in vivo*

**Figure 4:**
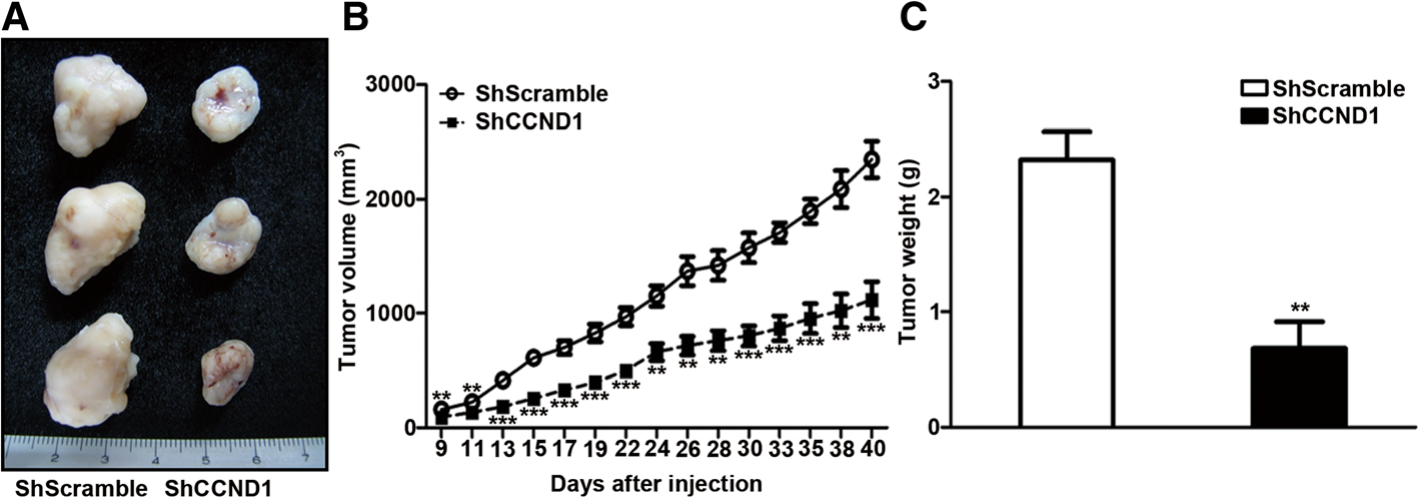
**Effect of ShCCND1 on tumor growth in the xenograft mouse model.** Gross observation of ShCCND1 xenografts showed a smaller volume compared to that of ShScramble xenografts **(A)**. Tumor volume of ShCCND1 xenografts was decreased compared to that of ShScramble xenografts during the experiment **(B)**. Tumor weight of ShCCND1 xenografts was significantly decreased compared to that of ShScramble xenografts **(C)**. The data presented are the mean ± SEM. **P < 0.01 and ***P < 0.001 versus the ShScramble-transduced cells.

ShCCND1 inhibited the expression of cyclin D1 and inhibited tumor cell growth *in vitro*. To further examine the inhibitory effect of ShCCND1 on tumor cell growth *in vivo*, a xenograft cancer model was used. As shown in Figure 4, tumors first appeared after 9 days, and tumor growth inhibition was observed throughout the entire experiment. At the end-point of the experiment, the difference was more evident; the last tumor volume was 2,348.2 ± 318.9 mm^3^ in the ShScramble group, whereas the volume in the ShCCND1 group was 1,115.0 ± 457.7 mm^3^ (P < 0.001). The average tumor weight in the ShScramble group was 2.3 ± 0.5 g compared to 0.6 ± 0.6 g in the ShCCND1 group. These data indicate that lentivirus-mediated ShCCND1 can inhibit tumor growth of NCI-N87 cells in nude mice.

### Intratumoral injection of lentivirus-mediated ShCCND1 attenuated the growth of established cancer

**Figure 5:**
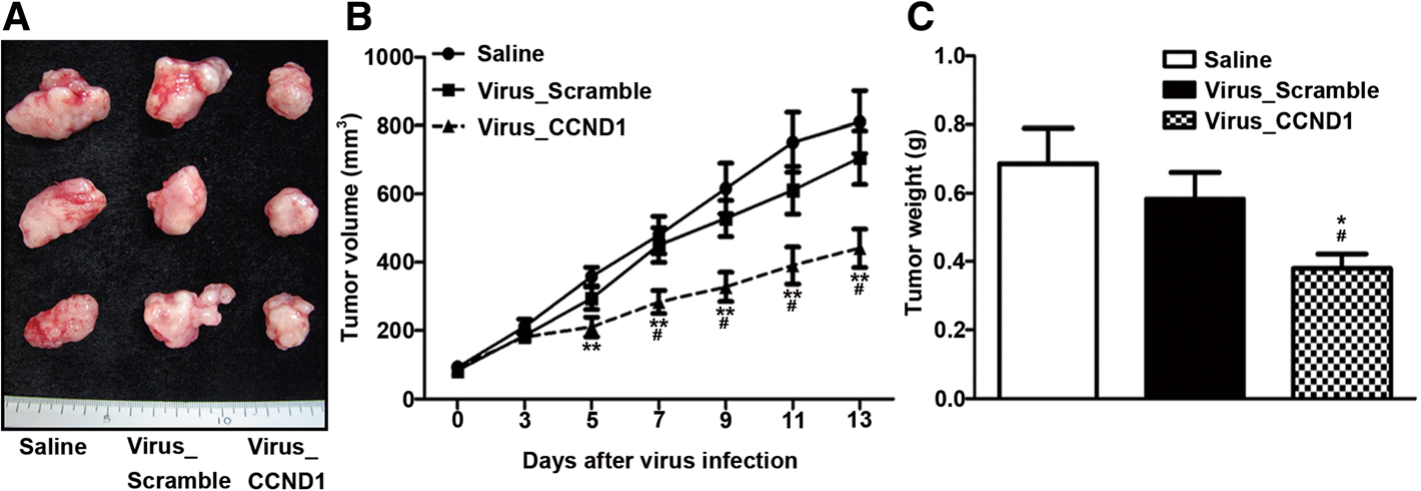
**Effect of intratumoral injection with Saline, Virus_Scramble, or Virus_CCND1 on established tumor growth.** Gross observation of Virus_CCND1 xenografts indicated a smaller volume than that of the Saline or Virus_Scramble xenografts **(A)**. Tumor volume of Virus_CCND1 xenografts was decreased compared to that of the Saline or Virus_Scramble xenografts during the experiment **(B)**. Tumor weight of Virus_CCND1 xenografts was significantly decreased compared to that of Saline or Virus_Scramble xenografts **(C)**. The data presented are the mean ± SEM. *P < 0.05 and **P < 0.01 versus Saline; ^#^P < 0.05 versus Virus_Scramble.

To determine whether the lentivirus could efficiently infect tumors and attenuate their growth, Virus_Scramble and Virus_CCND1 were infected into NCI-N87-derived cancer. Once tumors reached a volume of 80–100 mm^3^, the cancer was injected with 50 μl of a Saline, Virus_Scramble, or Virus_CCND1. The virus titer of Virus_Scramble and Virus_CCND1 was 4 × 10^7^ IU/ml. When experiments were terminated for tumor harvesting, the NCI-N87-derived cancer that was infected with Virus_CCND1 exhibited a 45.6% (P < 0.01) and a 37.5% (P < 0.05) decrease in volume by comparison with Saline and Virus_Scramble, respectively (Figure 5). Furthermore, the weight of Virus_CCND1-derived cancer was decreased by comparison with Saline and Virus_Scramble. The NCI-N87-derived cancer that was infected with Virus_CCND1 exhibited a 44.7% (P < 0.05) and a 34.9% (P < 0.05) decrease in weight, by comparison with Saline and Virus_Scramble, respectively. A statistically significant difference in cancer volumes between Saline- and Virus_CCND1-injected cancers was seen as early as five days after the viral injection (P < 0.01). Seven days after virus injection, there was a significant difference in cancer volume between Virus_Scramble and Virus_CCND1 (P < 0.05). Moreover, the subsequent growth of all Virus_CCND1-injected tumors remained significantly attenuated.

### Intratumoral injection of lentivirus-mediated ShCCND1 inhibited proliferation and apoptosis in pre-existing tumors

**Figure 6:**
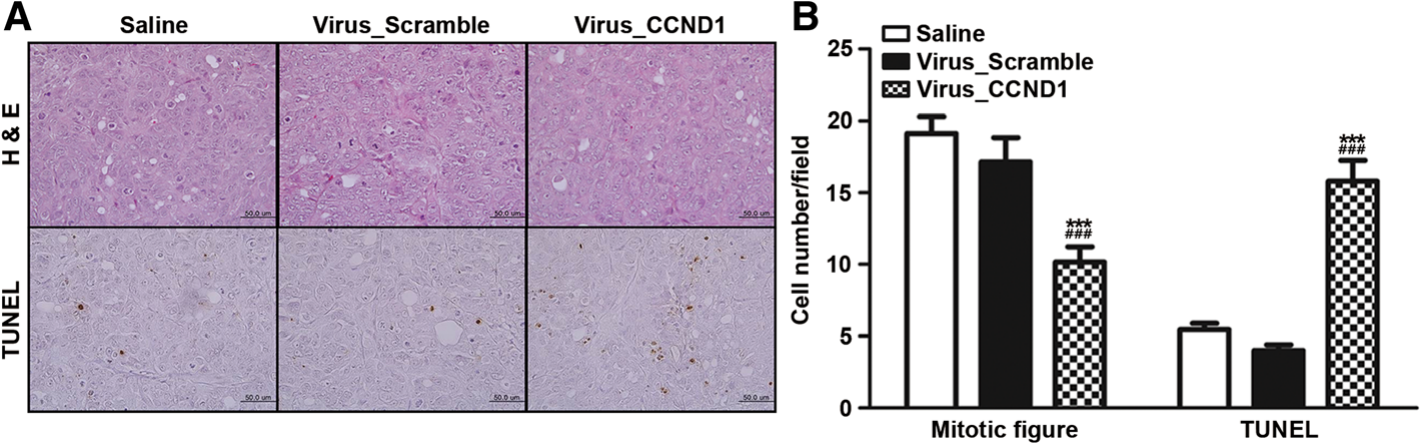
**Effect of intratumoral injection with Saline, Virus_Scramble, or Virus_CCND1 on proliferation and apoptosis in tumors.** In H&E staining, tumor tissues of Virus_CCND1 had decreased mitotic figure compared to that of Saline and Virus_Scramble, respectively. In the TUNEL assay, the number of apoptotic cells of Virus_CCND1 were increased compared to the number of Saline and Virus_Scramble **(A** and **B)**. Original magnification, 400×. The data presented are the mean ± SEM. ***P < 0.001 versus Saline and ^###^P < 0.001 versus Virus_Scramble.

To determine the biological effect of Virus_CCND1, xenograft sections were subjected to H&E or TUNEL staining. Treatment with Virus_CCND1 resulted in a significant decrease in the mitotic figure count compared to Saline and Virus_Scramble (Figure 6, P < 0.001). There was a significant induction in the mean number of TUNEL-positive cells in Virus_Scramble compared to the Saline and Virus_Scramble (P < 0.001). Taken together, these data suggest that targeting cyclin D1 with lentivirus-mediated ShCCDN1 could have an inhibition effect on cell proliferation and an induction effect of apoptosis *in vivo* on human gastric cancer.

## Discussion

Cyclin D1 is one of the three D-type cyclins and has been implicated in neoplastic function. The gene for cyclin D1 is located on chromosome 11q13 and is related to various cancers [20,21]. The increased expression of cyclin D1 has been associated with decreased G1 phase distribution and with decreased survival in many common cancers including gastric cancer [22-25]. Inhibition of cyclin D1 expression markedly suppressed cell proliferation and the beginning of S phase in the cell cycle of many cancers [25,26].

RNAi has been used as a therapeutic tool [27]. However, it is important to determine the most efficient and straightforward delivery strategy of the siRNA. The present study highlights the use of the shRNA-based technique, specifically the use of shRNA-based recombinant lentiviral vector to produce siRNA [3,28].

Lentiviruses are in the genus of the retroviridae family, which is an efficient vehicle for gene transduction and integration. In contrast to other viral vectors, lentiviral vectors have many advantageous. They have a lower immunogenicity, decreased likelihood of insertion mutagenesis, the ability to efficiently infect both primary and non-dividing cells, and a high capability of transgene insertion. In addition, they have been used as efficient preclinical models [14,29]. Therefore, they are ideally suited as a silencing technique in human cancer cells [11].

In this study, ShCCND1 resulted in stable integration of virus into the cellular genome and marked inhibition of cyclin D1 protein levels. Our previous study also reported that ShCCND1 significantly inhibited the expression levels of cyclin D1 and pRB, the major downstream molecule of cyclin D1, in another gastric cancer cell, AGS [30]. Downregulated of cyclin D1 was related to decreased cell proliferation, colony formation potential, and cell motility *in vitro*. Downregulation of cyclin D1 was associated with both the cell cycle and apoptosis; therefore, ShCCND1 significantly induced apoptosis and G1 arrest. G1 phase arrest allows cells to either undergo repair or enter the apoptotic pathway to maintain tissue homeostasis and eliminate the mutated neoplastic and hyperproliferating neoplastic cells from the system. In recent years, the concept of cell cycle regulation-mediated apoptosis has gained increasing attention, and it has emerged as a potential way to inhibit cell growth [3,31]. As such, ShCCND1 may be a potent therapeutic agent by causing G1 phase arrest and inducing apoptosis [32]. Collectively, these results clearly demonstrate that the anticancer effect of ShCCND1 NCI-N87 cells is mediated by induction of cell cycle arrest and apoptosis.

To analyze the therapeutic potential of the lentivirus expressing ShCCND1, *in vivo* experiments were performed. The recombinant lentivirus including ShCCND1 was intratumorally injected into established tumors. Intratumoral injection of the recombinant lentivirus suppressed the growth of established tumors arising from NCI-N87 cells. This result demonstrates that downregulation of cyclin D1 by the lentivirus-mediated shRNA strategy resulted in a prolonged biological effect *in vivo*. These results were also supported by the H&E and TUNEL staining data. Mitotic figure is a marker of proliferation and TUNEL-positive staining is a marker of apoptosis; results from these experiments suggested that silencing of cyclin D1 inhibited cancer cell growth *in vivo*. The decrease of mitotic figure count and increase in the number of TUNEL-positive cells are consistent with the known effects of silencing cyclin D1 and with the *in vitro* data presented in this study.

There are several reasons that intratumoral injection of lentivirus-mediated ShCCND1 may have diverse advantageous effects. Targeting cyclin D1 could hinder progress toward advanced cancer because cyclin D1 levels increase in the relatively early stages of tumorigenesis [33]. Furthermore, cyclin D1 is an essential component for carcinogenesis in some cancers, which is supported by the fact that cyclin D1 knockout mice do not make mammary carcinomas in spite of the overexpression of c-*neu* and v-Ha-*ras*[34]*.* Gastric cancer is characterized by the interaction between cancer cells and stromal cells that contain proliferating cancer-associated activated fibroblasts, endothelial cells, and inflammatory cells. These stromal cells have been suggested to confer a growth advantage to gastric cancer cells within the tumor mass [35,36]. Direct injection of Virus_CCND1 into gastric cancer would result in a downregulation of cyclin D1 not only in cancer cells but also in non-cancerous stromal cells. Therefore, direct injection of Virus_CCND1 effectively inhibited gastric cancer growth due to growth inhibition by simultaneously blocking cancer cell and non-cancer cell growth. While intravenous injection of lentivirus showed histological damage to the liver cells, intratumoral injection predominantly infected the tumor instead of the liver and other organs. In view of this fact, intratumoral injection of the lentiviral vector is more advantageous than intravenous injection [37]. Intratumoral injection of lentivirus should be generally applicable to human solid tumors [38]. Therefore, direct injection of lentivirus-mediated ShCCND1 is an efficient tool to significantly suppress cyclin D1 in gastric cancer cells. But because this study has some limitations, such as off-target effect or side-effect of lentivirus-mediated ShCCND1 *in vivo*, further study will be necessary to elucidate the accurate balance between therapeutic efficacy and safety of lentivirus-mediated ShCCND1 [39].

## Conclusion

Taken together, intratumoral injection of lentivirus-mediated ShCCND1 could efficiently inhibit gastric cancer growth *in vitro* and *in vivo*. These results also present the exciting possibility that this lentivirus-mediated ShCCND1 could ultimately be delivered into established tumors of gastric cancer patients by endoscopic ultrasonography, thereby producing a new therapeutic strategy for advanced gastric cancer. The silencing efficiency of shRNA varies based on different shRNA sequences, shRNA expression cassettes, and cancer cell lines [40,41], which is a disadvantage because it requires comprehensive bioinformatic and experimental data to select the most effective shRNA against a particular target. Therefore, these experimental results suggest the possibility that lentivirus-mediated shRNA targeting of cyclin D1 can be used as a new gastric cancer therapy.

## Abbreviations

ShSramble: ShRNA-targeting scramble; ShCCND1: ShRNA sequence targeting cyclin D1; PI: Propidium iodide; IU: Infectious unit; Virus_Scramble: Virus containing ShScramble; Virus_CCND1: Virus containing ShCCND1.

## Competing interests

The authors declare that they have no competing interests.

## Authors’ contributions

JHS carried out *in vivo* and *in vitro* experiments, participated in the design of the study and drafted the manuscript. ESJ carried out in vivo experiments and critically revised the manuscripts. YKC participated in the study supervision and critically reviewed the manuscript. All authors read and approved the final manuscript.

## Pre-publication history

The pre-publication history for this paper can be accessed here:

http://www.biomedcentral.com/1471-2407/14/175/prepub
